# The high risk of malarial recurrence in patients with *Plasmodium*-mixed infection after treatment with antimalarial drugs: a systematic review and meta-analysis

**DOI:** 10.1186/s13071-021-04792-5

**Published:** 2021-05-25

**Authors:** Aongart Mahittikorn, Frederick Ramirez Masangkay, Kwuntida Uthaisar Kotepui, Giovanni De Jesus Milanez, Manas Kotepui

**Affiliations:** 1 Department of Protozoology, Faculty of Tropical Medicine, Mahidol University, Bangkok, Thailand; 2 Department of Medical Technology, Institute of Arts and Sciences, Far Eastern University-Manila, Manila, Philippines; 3 Medical Technology, School of Allied Health Sciences, Walailak University, Tha Sala, Nakhon Si Thammarat, Thailand

**Keywords:** *Plasmodium*, Malaria, Mosquito, Artemisinin, Chloroquine, Treatment failure, Relapse

## Abstract

**Background:**

Malaria mixed infections are often unrecognized by microscopists in the hospitals, and a delay or failure to treat *Plasmodium*-mixed infection may lead to aggravated morbidity and increased mortality. The present study aimed to quantify the pooled proportion and risk of malarial recurrences after the treatment of *Plasmodium*-mixed infection. The results of the study may provide benefits in the management of *Plasmodium*-mixed infection in co-endemic regions.

**Methods:**

This systematic review and meta-analysis searched the international Prospective Register of Systematic Reviews (PROSPERO; ID = CRD42020199709), MEDLINE, Web of Science, and Scopus for potentially relevant studies in any language published between January 1, 1936, and July 20, 2020, assessing drug efficacy in patients with *Plasmodium*-mixed infection. The primary outcome was the pooled prevalence of *Plasmodium* parasitemia after initiating antimalarial treatment for *Plasmodium*-mixed infection. The secondary outcome was the pooled risk ratio (RR) of malarial recurrence in *Plasmodium*-mixed infection compared with those in *Plasmodium falciparum* and *Plasmodium vivax* mono-infection. The pooled analyses were calculated by random-effects meta-analysis. After the initial treatment in different days of recurrences (≤ 28 days or > 28 days), the risk of *Plasmodium* parasitemia was compared in subgroup analysis.

**Results:**

Out of 5217 screened studies, 11 were included in the meta-analysis, including 4390 patients from six countries. The pooled prevalence of all recurrences of *Plasmodium*-mixed parasitemia was 30% (95% confidence interval (CI) 16–43; *I*^2^: 99.2%; 11 studies). The RR of malarial recurrence within 28 days after the initial treatment (clinical treatment failure) of *Plasmodium*-mixed parasitemia compared with the treatment of *P. falciparum* was 1.22 (*p*: 0.029; 95% CI 1.02–1.47; Cochran Q: 0.93; *I*^2^: 0%; six studies), while there was no significant difference in the risk of recurrence 28 days after initial treatment compared with the treatment of *P. falciparum* (*p*: 0.696, RR: 1.14; 95% CI 0.59–2.18; Cochran Q < 0.05; *I*^2^: 98.2%; four studies). The subgroup analysis of antimalarial drugs showed that significant malarial recurrence within 28 days was observed in patients treated with artemisinin-based combination therapies (ACTs) with no significant heterogeneity (*p*: 0.028, RR: 1.31; 95% CI 1.03–1.66; Cochran Q: 0.834; *I*^2^: 0%).

**Conclusions:**

The present findings showed a high prevalence of malarial recurrence after the initial treatment of *Plasmodium*-mixed infection. Moreover, significant malaria recurrence of mixed infection occurred within 28 days after treatment with ACTs.

**Graphic Abstract:**

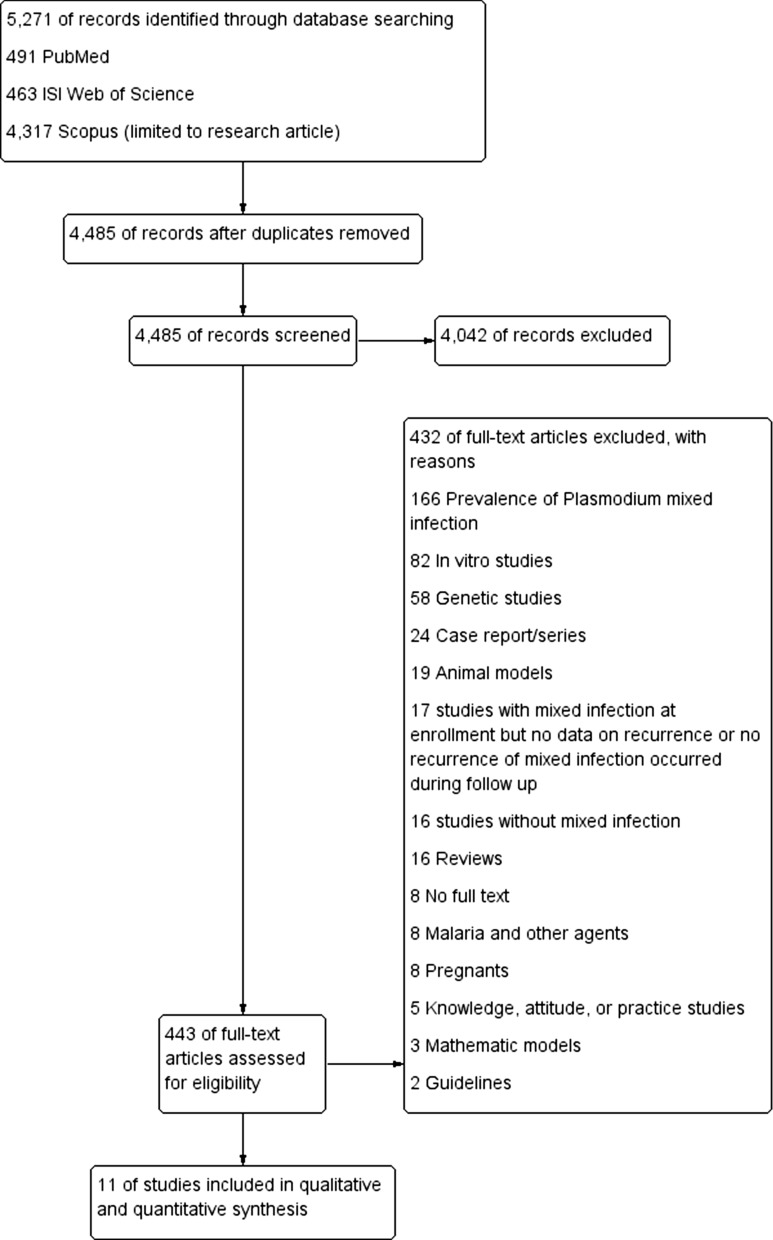

**Supplementary Information:**

The online version contains supplementary material available at 10.1186/s13071-021-04792-5.

## Background

Malaria remains important to global health, as it is related to severe disease morbidity and mortality [1]. Although five species of *Plasmodium* are recognized as the cause of malarial diseases in humans, *Plasmodium falciparum* and *Plasmodium vivax* are the most common *Plasmodium* species infecting humans worldwide [1–3]. *P. falciparum* infection is the most common cause of death from malaria, particularly in endemic areas of stable transmission or high malaria endemicity [3, 4]. *P. vivax* is the most common cause of benign malaria in Central America, South America, and Asia [5–8]. However, severe malaria and poor outcome in patients with *P. vivax* infection can occur [9, 10]. *P. malariae* and *P. ovale* are recognized as benign malaria parasites, but may cause severe malaria [11]. *P. ovale* parasites are divided into *P. ovale curtisi* and *P. ovale wallikeri* according to the dimorphism in defined genes [12, 13].

The coexistence of two or more *Plasmodium* species in a single-host or mixed-species infection can occur in an endemic area [14], and this has disrupted the diagnosis and treatment of malaria. A previous study suggested that *Plasmodium*-mixed infection may be acquired by simultaneous inoculation of sporozoites from multiple infected anopheline mosquitoes [15]. Although the interaction of two *Plasmodium* species in a single host remains controversial, a previous study demonstrated that a mixed infection of *P. falciparum* and *P. vivax* in a single human host can exhibit clinical signs in two ways: (1) suppressing each other and therefore reducing the severity of malaria, or (2) enhancing each other and therefore leading to an increased risk of severe malaria [16]. Mixed infections are often unrecognized by microscopists in hospitals. The sensitivity of microscopic observation of stained thick and thin blood films, as the gold standard for malaria parasite detection, is too low to detect *Plasmodium*-mixed species due to very low parasitemia in mixed infections. Recently, the polymerase chain reaction (PCR) method has been conducted to detect *Plasmodium* mono-infection [17] and *Plasmodium*-mixed infection with high evidence of sensitivity and specificity for detecting low parasitemia [18–20]. Furthermore, a delay or failure to treat *Plasmodium*-mixed infection may lead to aggravated morbidity and increased mortality rate. Therefore, the consideration of *Plasmodium*-mixed infection and its management have important clinical and therapeutic implications, particularly in patients who are co-infected with *P. falciparum* and *P. vivax*. The present study aimed to quantify the pooled proportion and risk of malarial recurrences after treatment of *Plasmodium*-mixed infection. The results of this study may provide benefits to the management of *Plasmodium*-mixed infection in co-endemic regions.

## Methods

The protocol was registered in the International Prospective Register of Systematic Reviews (PROSPERO) (ID = CRD42020199709).

### Search strategy

MEDLINE, Web of Science, and Scopus were searched for potentially relevant studies on drug efficacy in patients with *Plasmodium*-mixed infection in any language published between January 1, 1936, and July 20, 2020. The search strategy was used as previously described [21], with some modifications in search terms for the present study (Additional file 1: Table S1).

### Eligibility criteria

Studies in any language published between January 1, 1936, and July 20, 2020, were included in the analysis if they explicitly reported the presence of recurrent parasitemia with any *Plasmodium* species after treatment for *Plasmodium*-mixed infection. The following studies were excluded: studies reporting the prevalence of *Plasmodium*-mixed infection, in vitro studies, genetic studies, case reports or series, studies with animal models, reviews, studies on co-infection of malaria and other agents, studies where all data on recurrence could not be extracted, studies with pregnant women, and studies where full-text manuscripts were unavailable. Studies were selected by two authors (AM and MK), and discrepancies were resolved by a discussion with a third author (KUK). The results of this study were reported according to the preferred reporting items for systematic reviews and meta-analyses (PRISMA) guidelines [22].

### Data extraction

The data from the included studies were extracted independently by two authors (AM and MK); any discrepancies between them were resolved by discussion for consensus. The following details were extracted into a standardized pilot datasheet (Excel form) before further analysis: authors, year of publication, study site, year of the experiment, and information on patients, including age, gender, clinical signs, number of patients with recurrence, number of patients with *Plasmodium*-mixed infection at baseline, number of patients with *Plasmodium falciparum* and *Plasmodium vivax* infections at baseline, day of recurrence after the initial treatment, antimalarial drugs used, and number of any *Plasmodium* species after treatment. Antimalarial drugs used in the included studies were categorized as artemisinin-based combination therapies (ACTs) or chloroquine for subgroup analysis. Based on 2015 [23] and 2021 [24] World Health Organization (WHO) guidelines for the treatment of malaria, recurrence was defined as (1) recurrence within 4 weeks of treatment, which was considered as a “treatment failure,” and (2) recurrence of fever and parasitemia more than 4 weeks after treatment, which may be due to either recrudescence or new infection. Therefore, the recurrence data extracted in this study were grouped into “recurrence within 28 days” and “recurrence after > 28 days”. The upper time limit of the definition of recurrence for people with multiple malaria episodes was up to 365 days from treatment.

### Quality of the included studies

The quality of the included studies was assessed using a tool developed by the Joanna Briggs Institute (checklist for quasi-experimental studies) [25].

### Statistical analysis

The pooled prevalence of recurrence of any *Plasmodium* parasitemia after treatment was estimated using random-effects meta-analysis with proportions pooled using the Freeman-Tukey double arcsine transformation. The risk ratio (RR) of malarial recurrence after treatment of *Plasmodium*-mixed infection compared with those after treatment of *P. falciparum* and *P. vivax* were estimated using random-effects meta-analysis. The subgroup analysis of days of recurrence (≤ 28 days and > 28 days) was conducted to determine whether recurrence was caused by clinical treatment failure or by other causes. The subgroup analysis of antimalarial drugs (ACTs or chloroquine) and clinical signs (severe or uncomplicated malaria) was also conducted. Data heterogeneity among the included studies was assessed using Cochran's heterogeneity statistic and quantified by the *I*^2^ statistic. All analyses were performed using Stata version 15 software (StataCorp LLC, College Station, TX, USA).

### Publication bias

Publication bias across the included studies was assessed using funnel plots and Egger’s test. In the funnel plot, the precision of the estimated intervention effect increases with the size of the study. Therefore, the effect estimates from small studies will scatter more widely at the bottom of the graph, with the spread narrowing among larger studies. Without bias, the plot should approximately resemble a symmetrical (inverted) funnel. For Egger’s test, the significance of the coefficients is based on a Student *t* distribution (*t* test) instead of the normal distribution (z-test). If a small-study effect was found by Egger’s test, the contour-enhanced funnel plot was further analyzed to explore the source of publication bias.

## Results

### Search results

**Fig. 1:**
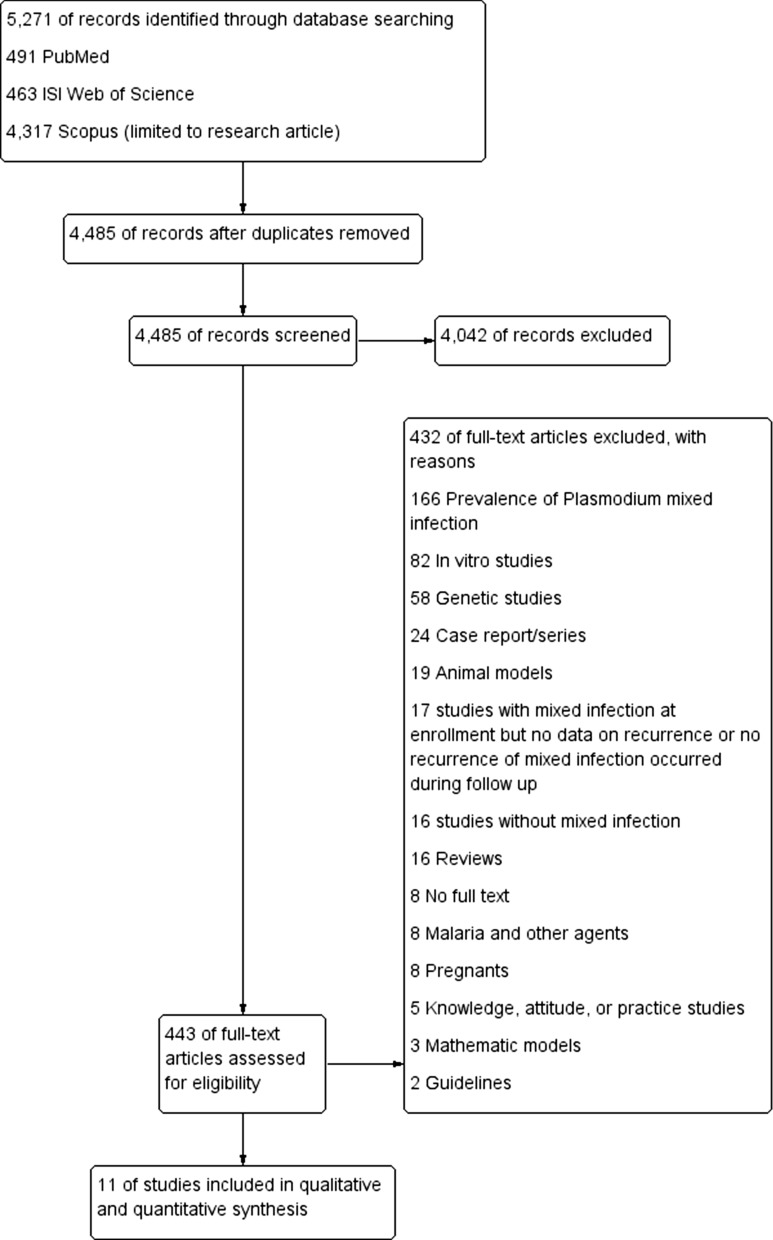
Flow chart for the study selection

After screening the titles and abstracts of 5271 studies published between January 1, 1936, and July 20, 2020, the full texts of 443 potentially relevant studies were reviewed. A total of 432 out of 443 studies did not meet the inclusion criteria and were excluded: 166 studies that reported the prevalence of mixed *Plasmodium* infection, 82 in vitro studies, 58 genetic studies, 24 case report/series, 19 animal models, 17 studies that reported mixed infection at enrollment but with no data on its recurrence during follow-up, 16 studies without mixed *Plasmodium* infection, 16 review articles, 8 studies with no full text, 8 studies that reported malaria and other pathogens, 8 studies in pregnant women, 5 knowledge/attitude/practice studies, 3 mathematic models, and 2 guidelines (Fig. 1). Eleven studies [26–36] were included in the present analysis (Table 1).

**Table 1:** Characteristics of the included studies

No.	Authors	Country	Year(s) of experiment	Study design	Follow-up period	Clinical signs	Antimalarial drugs	Number of patients with *Plasmodium*-mixed infection		Type of recurrence (≤ 28 days and > 28 days)	Species after treatment (n)	Number of patients with *P. falciparum* infection		Number of patients with *P. vivax* infection	
								Before treatment	After treatment			Before treatment	After treatment	Before treatment	After treatment
1	Ahmed et al. 2011	India	2007–2008	Observational study	28 days	Severe malaria	Intravenous quinine and oral quinine	6	23	Recurrence within 28 days	Pf + Pv	8	46	0	9
2	Dinko et al. 2013	Ghana	2010	Single-arm clinical trials	28 days	Uncomplicated malaria	ACTs (dihydroartemisinin–piperaquine)	4	14	Recurrence within 28 days (day 21)	Pf + Po (1), Pf + Pm (2), Pf + Pm + Po (1)	16	88	Not reported	Not reported
3	Douglas et al. 2011	Thailand	1991–2005	Observational study (16/24 RCT), single-arm clinical trials (8/24)	28 days (6 studies; 1398 patients), 42 days (11 studies; 5354 patients), or 63 days (7 studies; 3797 patients)	Uncomplicated malaria	ACTs (artesunate–atovaquone–proguanil, artemether, atovaquone–proguanil, artesunate, artesunate–tetracycline, artemether–lumefantrine, artesunate–mefloquine, quinine–mefloquine, quinine, quinine–tetracycline)	574	1164	Recurrence more than 28 days (by day 63)	Pf + Pv	2759	9385	Not reported	Not reported
4	Genton et al. 2005	Papua New Guinea	1994–1995	Single-arm clinical trials	28 days	Uncomplicated malaria	Amodiaquine (14 cases), chloroquine (1 case)	2	10	Recurrence within 28 days	Pf + Pv (9), Pf + Pv + Pm (1)	19	144	1	18
5	Lubis et al. 2020	Indonesia	2015	Single-arm clinical trials	42 days	Uncomplicated malaria	ACTs (dihydroartemisinin–piperaquine, artemether–lumefantrine)	2	39	1 recurrence within 28 days and 1 recurrence more than 28 days (day 35)	Pf + Pm (1), Pf + Pm + Pv (1)	1	114	Not reported	Not reported
6	Patriani et al. 2019	Indonesia	2004–2013	Observational study	365 days	Uncomplicated and severe malaria	Before 2006: quinine or chloroquine (69.2%), intravenous quinine (23.2%) In 2006: ACTs (96.6%), intravenous artesunate (19.3%) Single dose primaquine (23.5%), 14 days primaquine (70.3%)	166	1207	Recurrence more than 28 days (in 12 months)	Not specified	334	920	637	1334
7	Senn et al. 2013	Papua New Guinea	2006–2010	RCT	42 days	Uncomplicated malaria	ACTs (single dose of sulfadoxine/pyrimethamine- 3 days of amodiaquine/artesunate)	6	40	Recurrence more than 28 days (42 days)	Pf + Pv	31	372	84	634
8	Sikora et al. 2019	Indonesia	2004–2013	Observational study	28 days	Severe malaria	Intravenous artesunate and dihydroartemisinin–piperaquine, intravenous and oral quinine	81	1729	Recurrence within 28 days	Not specified	247	6915	88	1789
9	Siswantoro et al. 2006	Indonesia	2004–2005	Single-arm clinical trials	42 days	Uncomplicated malaria	Chloroquine- desethylchloroquine	10	15	Recurrence within 28 days (1 early treatment failure and 9 late treatment failure)	Pf + Pv	44	88	24	40
10	Smithuis et al. 2010	Myanmar	2008–2009	RCT	63 days	Uncomplicated malaria	ACTs (artesunate–amodiaquine, artemether–lumefantrine, fixed or loose artesunate–mefloquine, dihydroartemisinin–piperaquine)	95	129	Recurrence more than 28 days (by 63 days)		235	697	Not reported	Not reported
11	Sumawinata et al. 2003	Papua New Guinea	1995	Single-arm clinical trials	28 days	Uncomplicated malaria	Chloroquine	20	20	Recurrence within 28 days		52	55	24	29

*RCT* randomized control trial

### Characteristics of the included studies

Of the 11 included studies (Table 1), seven (63.6%) were conducted in the WHO South-East Asian Region, including Indonesia [30, 31, 33, 34], Thailand [28], India [26], and Myanmar [35] during 1991–2015. Three studies were conducted in Papua New Guinea during 1994–2010 [29, 32, 36], and one was conducted in Ghana during 2010 [27]. Two studies [26, 33] enrolled patients with severe malaria, while eight studies [27–30, 32, 34–36] enrolled patients with uncomplicated malaria. One study [31] enrolled patients with both severe and uncomplicated malaria in their study. The list of drugs for the treatment of malaria is shown in Table 1: one study [26] with intravenous quinine and oral quinine, six studies [27, 28, 30–32, 35] with ACTs, three studies [29, 34, 36] with chloroquine, and one study [33] with intravenous artesunate and dihydroartemisinin–piperaquine or intravenous and oral quinine. Most of the included studies (9/11, 81.8%) reported *Plasmodium*-mixed species after treatment of *Plasmodium*-mixed infection. One study [32] reported *P. falciparum* or *P. vivax* mono-infection, while another study [35] reported *P. vivax* mono-infection after treatment of *Plasmodium*-mixed infection. Recurrence caused by clinical treatment failure (≤ 28 days) was demonstrated in six studies [26, 27, 29, 33, 34, 36], while recurrence after treatment (> 28 days) was demonstrated in four studies [28, 31, 32, 35]. One study demonstrated recurrence on both ≤ 28 and > 28 days after treatment [30]. Assessment of risk of bias relating to individual studies is shown in Table 2.

**Table 2:** Assessment of the risk of bias in individual studies based on their quality using the JBI tool for quasi-experimental studies (non-randomized experimental studies)

No.	Authors	Cause and effect	Participants	Similar treatment/care	Controls	Multiple measurements of the outcome	Follow-up	Outcomes measured in the same way	Outcomes measured in a reliable way	Appropriate statistical analysis
1	Ahmed et al. 2011	Not applicable	Yes	Not applicable	Not applicable	Yes	Not applicable	Yes	Yes	Not applicable
2	Dinko et al. 2013	Yes	Yes	Yes	No	Yes	Yes	Yes	Yes	Yes
3	Douglas et al. 2011	Yes	Yes	Not applicable	Not applicable	Yes	Not applicable	Yes	Yes	Yes
4	Genton et al. 2005	Yes	Yes	Yes	No	Yes	Yes	Yes	Yes	Yes
5	Lubis et al. 2020	Yes	Yes	Yes	Yes	Yes	Yes	Yes	Yes	Yes
6	Patriani et al. 2019	Yes	Yes	Not applicable	Not applicable	Yes	Not applicable	Yes	Yes	Yes
7	Senn et al. 2013	Yes	Yes	Yes	No	Yes	Yes	Yes	Yes	Yes
8	Sikora et al. 2019	Yes	Yes	Not applicable	Not applicable	Yes	Not applicable	Yes	Yes	Yes
9	Siswantoro et al. 2006	Yes	Yes	Yes	No	Yes	Yes	Yes	Yes	Yes
10	Smithuis et al. 2010	Yes	Yes	Yes	Yes	Yes	Yes	Yes	Yes	Yes
11	Sumawinata et al. 2003	Yes	Yes	Yes	No	Yes	Yes	Yes	Yes	Yes

*JBI* Joanna Briggs Institute

### The pooled prevalence estimates of malarial recurrence

**Fig. 2:**
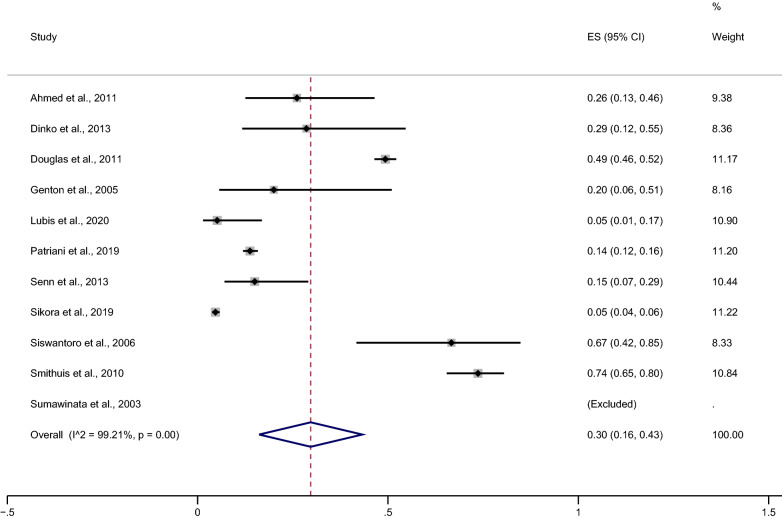
Pooled prevalence estimate of malarial recurrence

The prevalence of *Plasmodium* parasitemia was present in all 11 studies from six countries. The prevalence of all *Plasmodium* parasitemia after treatment ranged from 5–74%. The highest proportion of recurrence (74%; 95% CI: 65–80) was demonstrated in a study by Smithuis et al. [35], while the lowest proportion of recurrence was demonstrated in a study by Lubis et al. 2020 (5%; 95% CI 1–17) [30]. Overall, the estimated pooled prevalence of *Plasmodium* parasitemia after treatment of *Plasmodium*-mixed infection was 30% (95% CI 16–43; *I*^2^: 99.2%) (Fig. 2).

### The risk of *Plasmodium*-mixed species recurrence compared with *P. falciparum* recurrence

**Fig. 3:**
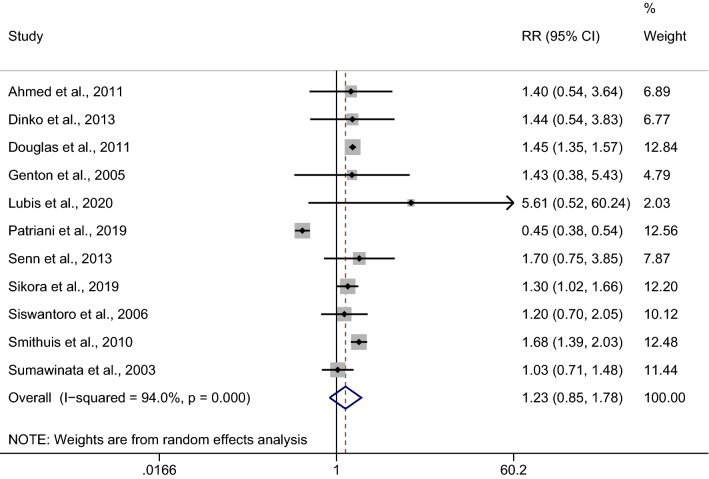
Estimated risk of recurrence in patients after treatment of *Plasmodium*-mixed infection compared to those after treatment of *P. falciparum*

The risk of malarial recurrence after treatment of *Plasmodium*-mixed infection was compared with malarial recurrence after treatment of *P. falciparum* infection. Overall, no significant difference in the risk of any recurrent parasitemia was observed when the recurrence risks from the 11 studies were pooled (*p*: 0.266, RR: 1.23; 95% CI 0.85–1.78; Cochran Q < 0.05; *I*^2^: 94%) (Fig. 3).

### Subgroup analysis of the days of recurrence

**Fig. 4:**
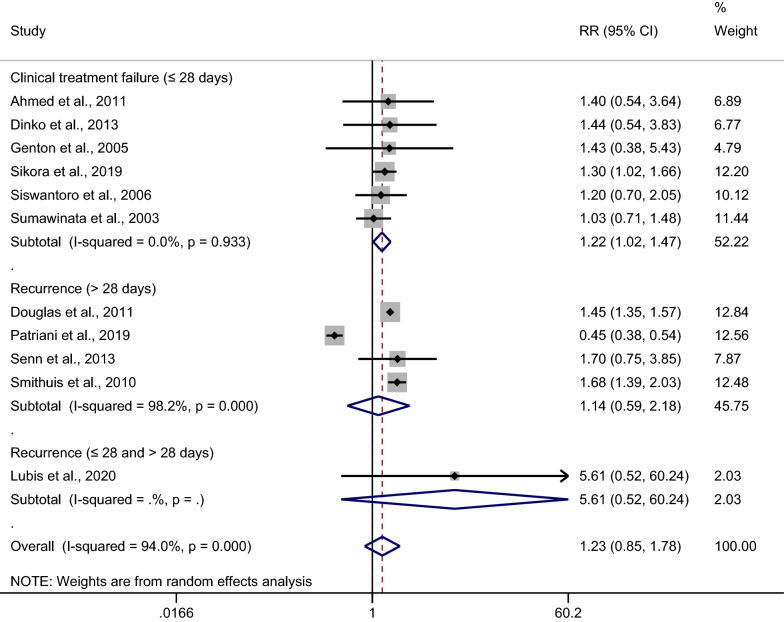
Subgroup analysis of recurrence in patients after treatment of *Plasmodium*-mixed infection

A subgroup analysis of the days of recurrence (≤ 28 days or > 28 days) was conducted for the 11 studies. A significantly higher risk of malarial recurrence within 28 days after the treatment of mixed *Plasmodium* infection was found, with no significant heterogeneity across the studies analyzed (*p*: 0.029, RR: 1.22; 95% CI 1.02–1.47; Cochran Q: 0.93; *I*^2^: 0%; six studies) (Fig. 4). A study by Sikora et al. in 2019 demonstrated a higher risk of malarial recurrence within 28 days after treatment (RR: 1.30; 95% CI 1.02–1.66). No significant difference was observed in the risk of malarial recurrence after 28 days of treatment for mixed *Plasmodium* infection, with significant heterogeneity across the studies analyzed (*p*: 0.696, RR: 1.14; 95% CI 0.59–2.18; Cochran Q < 0.05; *I*^2^: 98.2%; four studies). A higher risk of malarial recurrence after 28 days of treatment was demonstrated in the studies by Douglas et al. [28] and Smithuis et al. [35], while a lower risk of recurrence after 28 days of treatment was demonstrated in a study by Patriani et al. [31].

### Subgroup analysis of antimalarial drugs

**Fig. 5:**
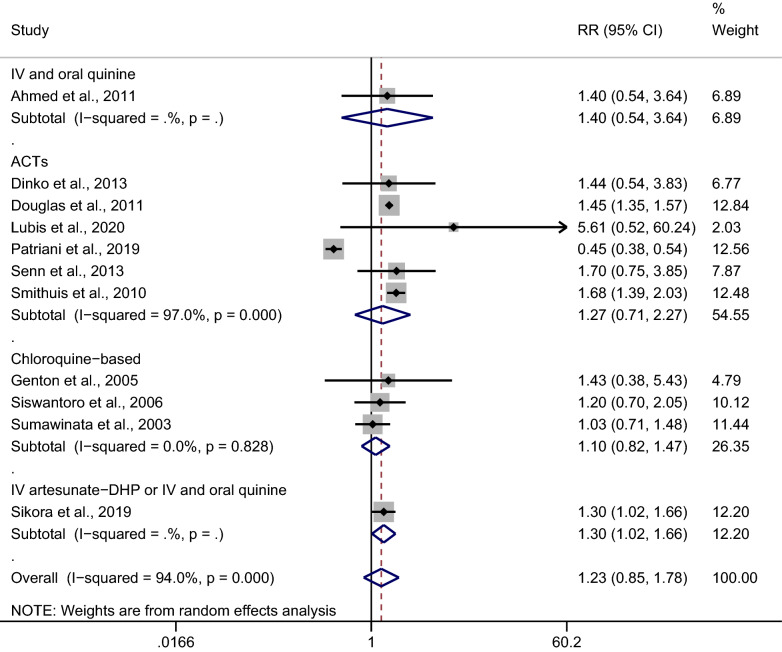
Subgroup analysis of antimalarial drugs used in patients with all recurrences. IV: intravenous

**Fig. 6:**
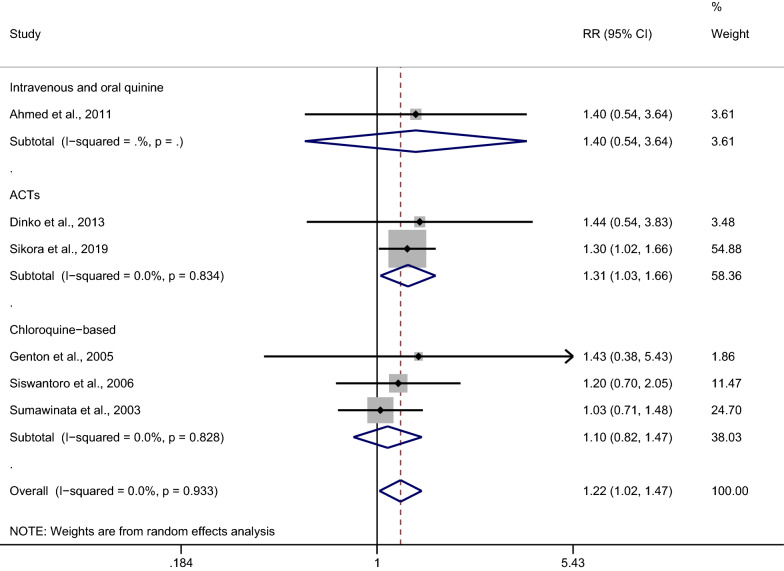
Subgroup analysis of antimalarial drugs used in patients with recurrence within 28 days

A subgroup analysis of antimalarial drugs was conducted using the data from 11 studies. Overall, no significant difference in the risk of malarial recurrence after treatment with ACTs was found between mixed *Plasmodium* infection and *P. falciparum* infection (*p*: 0.423, RR: 1.27; 95% CI 0.71–2.27; Cochran Q < 0.05; *I*^2^: 97%; six studies) (Fig. 5). No significant difference in the risk of malarial recurrence was found between *Plasmodium*-mixed infection and *P. falciparum* infection (*p*: 0.546, RR: 1.10; 95% CI 0.82–1.47; Cochran Q: 0.83; *I*^2^: 0%; three studies) after treatment with chloroquine. A subgroup analysis of antimalarial drugs was further performed in six studies [26, 27, 29, 33, 34, 36] which reported the recurrence of all *Plasmodium* parasitemia within 28 days after treatment. The results showed that the malarial recurrence within 28 days was significantly observed in patients treated with ACTs, with no significant heterogeneity (*p*: 0.028, RR: 1.31; 95% CI 1.03–1.66; Cochran Q: 0.834; *I*^2^: 0%), while there was no significant difference in the risk of malarial recurrence within 28 days in patients treated with chloroquine (*p*: 0.546, RR: 1.10; 95% CI 0.82–1.47; Cochran Q: 0.828; *I*^2^: 0%) (Fig. 6).

### Subgroup analysis of clinical signs

**Fig. 7:**
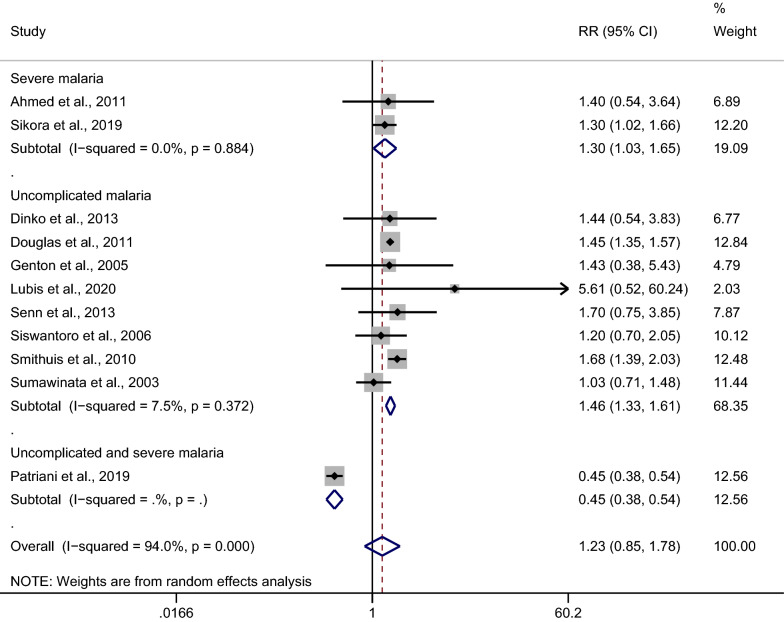
Subgroup analysis of clinical signs in patients with all recurrences

A subgroup analysis of clinical signs (severe or uncomplicated malaria) was conducted. The results showed that a significantly higher risk of malarial recurrence after treatment of *Plasmodium*-mixed infection was observed in patients with severe malaria, with no significant heterogeneity across the studies analyzed (*p*: 0.029, RR: 1.30; 95% CI 1.03–1.65; Cochran Q: 0.88; *I*^2^: 0%; two studies). In addition, a significantly higher risk of malarial recurrence after treatment of *Plasmodium*-mixed infection was also observed in patients with uncomplicated malaria, with no significant heterogeneity across the studies analyzed (*p* < 0.001, RR: 1.46; 95% CI 1.33–1.61; Cochran Q: 0.37; *I*^2^: 7.5%; eight studies) (Fig. 7).

### The risk of *Plasmodium*-mixed species recurrence compared with *P. vivax* recurrence

**Fig. 8:**
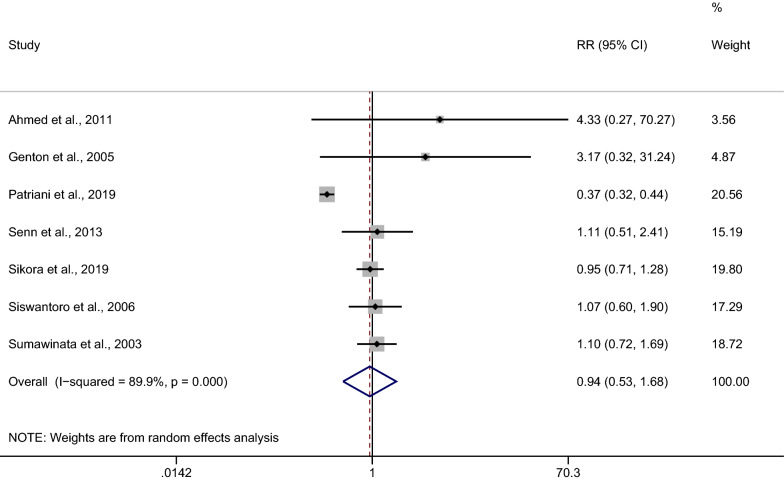
Estimated risk of recurrence in patients after treatment of *Plasmodium*-mixed infection compared to those after treatment of *P. vivax*

The risk of malarial recurrence after treatment of *Plasmodium*-mixed infection was compared with malarial recurrence after treatment of *P. vivax* infection. Overall, no significant difference in the risk of any recurrent parasitemia was observed when the recurrence risks from the seven studies were pooled (*p*: 0.847, RR: 0.94, 95% CI 0.53–1.68, Cochran Q < 0.05, *I*^2^: 89.9%) (Fig. 8).

### Publication bias

**Fig. 9:**
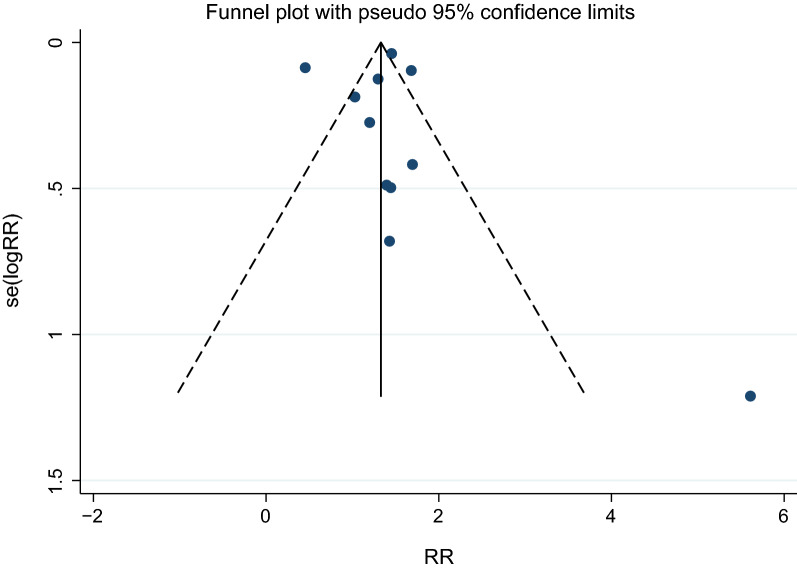
Funnel plot analysis

The publication bias is shown in Fig. 9, in which the dispersion of the nine-point estimates was close to the true intervention or pooled estimate. This result indicates the symmetry of the funnel plot. However, two studies exhibited a wide dispersion of the point estimates from the center, indicating the possibility of publication bias among the included studies. The possibility of publication bias due to smaller studies was further investigated by Egger’s test. The results of Egger’s test demonstrated that no small-study effects were found (*p*: 0.934; *t*: 0.09; coefficients: 0.15; standard error: 1.78), indicating that the publication bias might be due to other causes, such as the variability in included studies, especially concerning the prevailing *Plasmodium* species and resistance pattern driven by drug pressure.

## Discussion

Infection by *Plasmodium*-mixed species, if left untreated or managed poorly, can lead to severe malaria [37]. A previous systematic review and meta-analysis demonstrated that either *Plasmodium*-mixed infection or *P. falciparum* mono-infection showed a similar trend of complications in which severe anemia, pulmonary failure, and renal impairment were the three most common complications found [37]. The present study found a high estimated prevalence of *Plasmodium* parasitemia after treatment of *Plasmodium*-mixed infection (30%). This result indicated that treatment failure, relapse, or recrudescence might have occurred in these patients. The present study also demonstrated clearly that a significantly higher risk of malarial recurrence occurred within 28 days after treatment of *Plasmodium*-mixed infection compared to those with *P. falciparum* infection, while no significant recurrence was observed between the two groups after 28 days. This implies that the cause of malarial recurrence in patients with mixed infection might be due to early or late clinical treatment failure. In comparison to the treatment of *P. falciparum*, the treatment of *Plasmodium*-mixed infection significantly led to malaria recurrence within 28 days as observed in patients treated with ACTs, while no significant malarial recurrence within 28 days in patients treated with chloroquine was observed. This indicated that patients with *Plasmodium*-mixed infection who are treated with ACTs similar to the treatment of patients with *P. falciparum* can still experience treatment failure, particularly patients with severe malaria. This result was supported in studies by Sikora et al. [33] and Ahmed et al. [26], which reported that malarial recurrence was caused by clinical treatment failure among patients with severe malaria. Nevertheless, after treatment of *Plasmodium*-mixed infection, malarial recurrence can occur in patients with uncomplicated malaria, as demonstrated in the subgroup analysis of clinical signs. The risk of malarial recurrence was not observed in patients with mixed infections compared to those with *P. vivax* mono-infection. The significance of malarial recurrence in patients with uncomplicated malaria was observed in studies by Douglas et al. in Thailand [28] and Smithuis et al. in Myanmar [35]. This result indicated that the recurrence of malaria after treatment of *Plasmodium*-mixed infection might be caused by many factors, including drug resistance or inappropriate antimalarial drugs [23], severity signs, lack of malarial immunity [38, 39], or no additional dose of primaquine to reduce potential *P. vivax* transmission substantially [35].

Treatment decisions on malaria infection are different based on *Plasmodium* species and disease severity. In uncomplicated malaria, chloroquine and primaquine drugs are administered to treat *P. vivax*, while ACTs are administered to treat *P. falciparum* malaria since *P. falciparum* is resistant to chloroquine [23]. For the treatment of *Plasmodium*-mixed species, WHO suggested that ACTs (except artesunate + sulfadoxine–pyrimethamine) are effective against all malaria species and are the treatment of choice against mixed infection in co-endemic areas of *P. falciparum* and *P. vivax* [23]. In all severe malaria cases, intravenous artesunate or quinine is administered for at least 24 h before patients can tolerate oral medication [23]. Since misdiagnosis of mixed species of malaria infection might lead to severe malaria [37], intravenous artesunate or quinine was used to treat severe mixed malaria infection in two of the included studies [26, 33].

Most of the studies included in this meta-analysis were performed on the Asian continent because of the emergence of multidrug resistance in driving the treatment policy changes, but fewer malaria treatment options are available in Asian countries. It was also due to the potential spread of ACT resistance in sub-Saharan Africa because of the progression of chloroquine resistance and sulfadoxine–pyrimethamine resistance from Asia to sub-Saharan Africa in the past, which contributed to millions of childhood deaths [40, 41]. Previous studies reported the incidence of *P. vivax* parasitemia after the treatment of *P. falciparum* or mixed *Plasmodium* species in Southeast Asia [21, 28, 42]. Therefore, the efficacy of antimalarial treatment to prevent the recurrence of *P. vivax* parasitemia is an important consideration for clinical drug trials of malaria control strategies in this region.

This study had limitations. First, there was a limited number of studies reporting the treatment of mixed *Plasmodium* infection, which might result in a low statistical power to be applied over a large population. Some potentially eligible studies did not report the exact number of *Plasmodium* recurrences after treatment with antimalarial drugs, and no studies reported in non-English language were found for the present study, leading to a limited number of included studies. Second, the baseline characteristics of patients, such as age, could not be extracted in all the included studies since the treatment efficacy of malaria was improved as the age increased [43]. Therefore, the subgroup analysis and meta-regression analysis of age as a confounder for risk of recurrence could not be performed. Third, looking at the range of values for recurrence reported (5–74%), it is likely that the differences in human, vector, and parasite dynamics, study methodology, follow-up time, and treatment efficacy may account for some of the variations in recurrence. Fourth, the “true” RR and funnel plots of this study may be misleading due to the variability in the included studies, especially concerning the prevailing *Plasmodium* species and resistance pattern driven by drug pressure. Therefore, there is a need for a careful interpretation of the RR of malarial recurrence in patients with mixed *Plasmodium* infection after treatment with antimalarial drugs. Fifth, the present meta-analysis included the publication of Douglas et al. [28] that pooled the findings of multiple studies that applied different study designs, different medications, and outcome assessment; in essence, this is a pooling of study findings but without the statistical benefits of a meta-analysis. This is akin to including a meta-analysis in the present meta-analytic data. Therefore, the interpretation of meta-analysis in the present study requires major attention for this included study.

## Conclusion

The present findings showed a high prevalence of malarial recurrence after initial treatment of *Plasmodium*-mixed species. Moreover, significant malaria recurrence of mixed infection occurred within 28 days after treatment with ACTs. Therefore, in regions where more than one *Plasmodium* species are endemic, the use of appropriate antimalarial drugs with increased vigilance is required and should be strengthened during and after treatment. Further research is required to gain a better understanding of the mechanisms involved in the recurrence observed after treatment with ACTs.

## Supplementary Information

**Additional file 1: Table S1.** Search terms.

## Data Availability

All data in the manuscript and supplementary files are available.

## Abbreviations

ACTs: Artemisinin-based combination therapies
CI: Confidence interval
PCR: Polymerase chain reaction
PRISMA: Preferred reporting items for systematic reviews and meta-analyses
RR: Risk ratio
WHO: World Health Organization
