# Supplementary material for: The high risk of malarial recurrence in patients with Plasmodium-mixed infection after treatment with antimalarial drugs: a systematic review and meta-analysis
Source: Parasit Vectors. 2021 May 25;14:280. doi: 10.1186/s13071-021-04792-5 (PMC8145851; doi:10.1186/s13071-021-04792-5)
Supplement: Supplementary file 1 — Additional file 1: Table S1. Search terms. [file 13071_2021_4792_MOESM1_ESM.docx]

**Table S1.** Search term.

| **Databases** | **Search terms** | **Date** |
| --- | --- | --- |
| PubMed | (Plasmodium OR malaria) AND mixed AND (primaquine OR allopurinol OR amodiaquine OR atovaquone OR artemisinin OR arteether OR artesunate OR artemether OR artemotil OR atovaquone OR azithromycin OR artekin OR chloroquine OR chlorproguanil OR cycloguanil OR clindamycin OR coartem OR dapsone OR dihydroartemisinin OR duo-cotecxin OR doxycycline OR halofantrine OR lumefantrine OR lariam OR malarone OR mefloquine OR naphthoquine OR naphthoquinone OR pafuramidine OR piperaquine OR primaquine OR proguanil OR pyrimethamine OR pyronaridine OR proguanil OR quinidine OR quinine OR riamet OR sulphadoxine OR sulfamethoxazole OR tetracycline OR tafenoquine) | 20 July 2020 |
| Scopus | (Plasmodium OR malaria) AND mixed AND (primaquine OR allopurinol OR amodiaquine OR atovaquone OR artemisinin OR arteether OR artesunate OR artemether OR artemotil OR atovaquone OR azithromycin OR artekin OR chloroquine OR chlorproguanil OR cycloguanil OR clindamycin OR coartem OR dapsone OR dihydroartemisinin OR duo-cotecxin OR doxycycline OR halofantrine OR lumefantrine OR lariam OR malarone OR mefloquine OR naphthoquine OR naphthoquinone OR pafuramidine OR piperaquine OR primaquine OR proguanil OR pyrimethamine OR pyronaridine OR proguanil OR quinidine OR quinine OR riamet OR sulphadoxine OR sulfamethoxazole OR tetracycline OR tafenoquine)  Search option: limit to research article | 20 July 2020 |
| ISI Web of Science | (Plasmodium OR malaria) AND mixed AND (primaquine OR allopurinol OR amodiaquine OR atovaquone OR artemisinin OR arteether OR artesunate OR artemether OR artemotil OR atovaquone OR azithromycin OR artekin OR chloroquine OR chlorproguanil OR cycloguanil OR clindamycin OR coartem OR dapsone OR dihydroartemisinin OR duo-cotecxin OR doxycycline OR halofantrine OR lumefantrine OR lariam OR malarone OR mefloquine OR naphthoquine OR naphthoquinone OR pafuramidine OR piperaquine OR primaquine OR proguanil OR pyrimethamine OR pyronaridine OR proguanil OR quinidine OR quinine OR riamet OR sulphadoxine OR sulfamethoxazole OR tetracycline OR tafenoquine)  Search option: All fields | 20 July 2020 |
